# Quality by Design for Preclinical In Vitro Assay Development

**DOI:** 10.1002/pst.2430

**Published:** 2024-09-24

**Authors:** Jonathan Jones, Bairu Zhang, Xiang Zhang, Peter Konings, Pia Hansson, Anna Backmark, Alessia Serrano, Ulrike Künzel, Steven Novick

**Affiliations:** ^1^ Data Sciences and Quantitative Biology, Discovery Sciences, R&D AstraZeneca Cambridge UK; ^2^ Data Sciences and Quantitative Biology, Discovery Sciences, R&D AstraZeneca Gothenburg Sweden; ^3^ Bioscience Cardiovascular, Research and Early Development, Cardiovascular, Renal and Metabolism (CVRM), BioPharmaceuticals R&D AstraZeneca Gothenburg Sweden; ^4^ Functional Genomics, Discovery Sciences, R&D AstraZeneca Cambridge UK; ^5^ Data Sciences and Quantitative Biology, Discovery Sciences, R&D AstraZeneca Gaithersburg Maryland USA

**Keywords:** assay optimization, CRISPR, design of experiments, design space, JMP, screening

## Abstract

Quality by Design (QbD) is an approach to assay development to determine the design space, which is the range of assay variable settings that should result in satisfactory assay quality. Typically, QbD is applied in manufacturing, but it works just as well in the preclinical space. Through three examples, we illustrate the QbD approach with experimental design and associated data analysis to determine the design space for preclinical assays.

## 1 Introduction

In the pharmaceutical industry, hit screening is the key part of the preclinical pipeline for finding drug targets or lead compounds. Due to the rapid development of technologies and computational tools, the capability of screening has increased dramatically in the past 10–20 years. Scientists are running extensive screens in the laboratories, from high throughput biochemical screens for testing millions of compounds [1] to more complex cell‐based clustered regularly interspaced short palindromic repeats (CRISPRs) screens for exploring the phenotype of thousands of genes [2]. Promising drugs identified and validated by in vitro assays are then further screened in animal experiments or other more sophisticated assays to explore their efficacy and toxicity. It is crucial for scientists to make accurate decision from hit screens, which requires the in vitro assays constructed and developed to be fit‐for‐purpose, precise, robust, and reproducible. At AstraZeneca, we develop preclinical assays using a Quality by Design (QbD) approach.

The principles of QbD have been introduced and accepted for the quality of pharmaceutical products in the manufacturing process [3, 4, 5, 6, 7]. QbD is a systematic framework where assay quality is embedded into the pharmaceutical development from the beginning of the process. Although there are published studies on the applications of QbD in the development of drug products [8, 9, 10], there is scant literature on its employment in the preclinical space [11, 12], implying that this approach has yet to be applied widely for in vitro assays for hit screening in drug discovery. During our work with assay developers and hit‐screening scientists, we noticed a lack of QbD guidelines and references for in vitro assay development in the drug discovery space. In this tutorial article, we illustrate the QbD framework that can be used for in vitro assay development and apply the concept to three different assay types as case studies.

In pharmaceutical development, the QbD framework combines prior knowledge and probability modeling together with results of studies using design of experiments (DoE) [3, 4]. DoE is a statistical approach to experimental design that aims to systematically and efficiently investigate the relationship between outcomes and factors of interest. Key characteristics of QbD relevant to in vitro assay development include identification of critical quality attributes (CQAs), which are characteristics to define a desired assay quality, and determination of critical process parameters (CPPs), which are assay variables that are known to affect assay quality as measured via the CQAs. Through carefully crafted DoE and appropriate analysis, the CQAs of an assay can be predicted across different CPP levels. An analysis of the study data should permit the identification of a multi‐dimensional region of CPP levels that result in acceptable CQA values, which forms the *design space* for assay. This crucial element of QbD, the design space, is defined as the multidimensional combination and interactions between CPPs and CQAs [3, 4]. It can be described as the ranges or levels of assay CPPs that should result in acceptable CQA values.

A QbD‐developed assay is of keen interested to hit‐screeners. Because the assets (compounds, genes, etc.) identified by the hit screen are further developed, it is paramount to build confidence in the assay results so that false positives (hits that are later invalidated) are not pursued at the next drug discovery step and false negatives (misses that would be hits in a sensitive experiment) are not overlooked. Prior to applying QbD to preclinical assay development, it was our experience that hit‐screening scientists were provided a single target setting for CPPs for running the assay. Regrettably, small mishaps often occurred in the assay procedure, such as inadvertent lengthening of incubation time or accidental increasing/decreasing of media concentrations, that yielded results of questionable quality. Our hit screening scientists were excited to learn that we could build a design space to provide a range of CPPs that assures a high probability of obtaining acceptable estimates of the underlying CQA value, meaning that small perturbations in the assay settings would not negatively affect confidence in the assay results.

For a simple thought experiment, consider the task of a baker who wishes to determine the conditions of oven temperature and cooking time for baking cookies. Assume that the cookie dough is pre‐made and pre‐apportioned and that any two equal‐sized portions of dough are interchangeable. The baker assembled a panel of judges to assess the taste of the cookies using a five‐point Likert scale (1 = bad to 5 = good). From experience, the baker determined that whenever *taste* ≥ 4, the quality of the cookies was sufficient. Thus, *taste* is assigned as the CQA with a quality cutoff of four and above. Further, the baker was curious to study the link between *temperature*, *time*, and *taste*, because those factors are thought to influence the quality of the cookies. It follows that *temperature and time* are the CPPs. Prior to experimentation, the baker had been carefully baking at a temperature of exactly 175°C (350 F) and a time of exactly 15 min; but, sometimes a bake‐shop employee would inadvertently change the oven temperature or lose track of time, resulting in cookies the baker was unsure could be sold. The baker wondered if the cookie batch quality would be acceptable if the temperature and/or time settings were changed purposely or accidentally. After statistically designing several studies (i.e., batches of cookies with different temperature and time settings) and based on the judges scoring, it was determined that a range of temperatures between 175°C (350 F) and 190°C (375 F) and a range of times between 12 and 15 min will reliably produce cookies with *taste* ≥ 4. That is, baking within the given ranges results in a high predictive probability that *taste* ≥ 4. By building quality into the experimental design, the design space is 175 ≤ *temperature* ≤ 190 and 12 ≤ *time* ≤ 15. The baker decided that, for future cookie batches, the target temperature will be set to 182°C (360 F) and the target time set to 13.5 min, with the knowledge that small deviations in temperature and time will not ruin the batch.

More details of the QbD process will be introduced in the Methods section of this tutorial article. Then, three case studies of different assay types will be introduced. In the first case study, the development of a cell‐based AlphaLISA assay is provided. The second case study involves a cell‐based time‐resolved cAMP biosensor assay. In the last case study, QbD is applied to a complex CRISPR assay. Each case study provides a different window into CPP and CQA selection as well as differences in experimental design and data analysis.

This tutorial article is intended as a reference for both statisticians, who are interested in the application of QbD, and assay developers, who are conducting experiments in the laboratories. Therefore, in this article we will introduce both basic statistical methods, which can be easily performed in interface‐based statistical software such as JMP, and more advanced statistics methods that requires coding skills in R software.

## 2 Methods

### 2.1 Overview of QbD for In Vitro Assay Development

**FIGURE 1:**
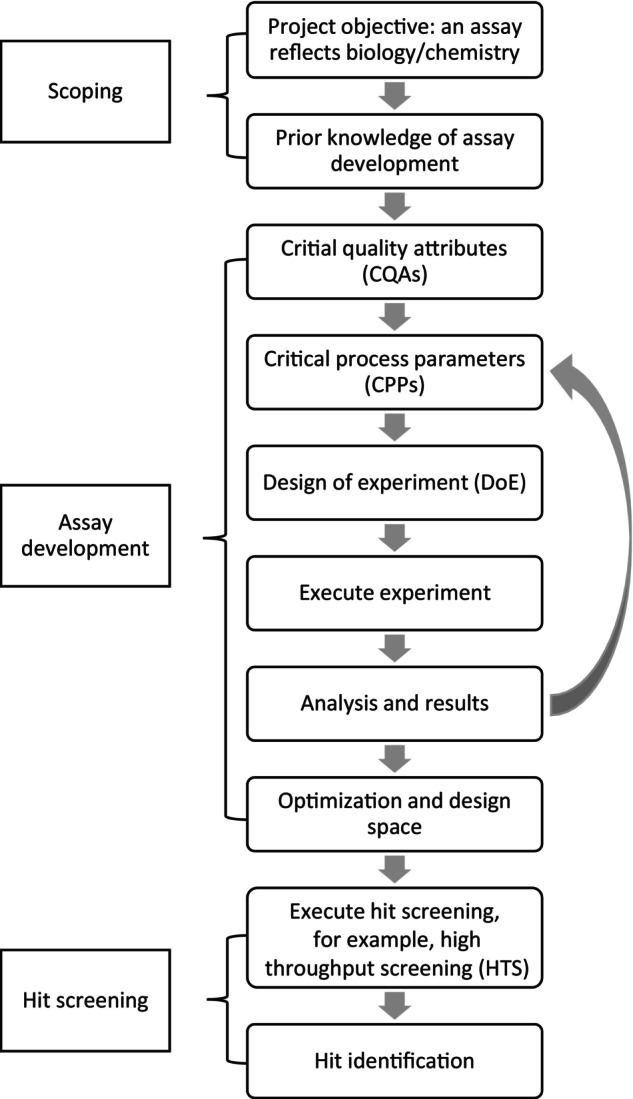
Flow chart of the QbD process.

Originally, the QbD approach was proposed for developing formulations of pharmaceutical products and manufacturing processes [3, 4, 5, 6, 7]. Fundamentally, the concept is that a higher product performance can be achieved through control of critical sources of variability in the design [13]. Although the essence of QbD is applicable to in vitro assay development, the processes require some modifications. A typical flow chart of QbD modified for in vitro assay development is shown in Figure 1.

#### 2.1.1 Scoping Phase

In a large pharmaceutical company, there can be hundreds to thousands of in vitro assays running each year. Usually, a project starts with the researchers' scientific ideas and then the assay is built to address biological or chemical questions. Prior to any investment, multiple rounds of assessment and discussion may happen, such as on topics of cost–benefit, assay feasibility, and risk. During this stage, scientists build and share knowledge through the systematic review of literature or collecting information from previous experiments of similar assays. It is important for the project team to have clear objectives and further validation plan at the beginning of a project. For example, plate‐based arrayed CRISPR genetic assays are often used at AstraZeneca for identifying and validating drug targets based on multiple phenotypical and molecular markers [14]. Arrayed CRISPR screens can either be performed at genome‐wide scale (~20,000 genes) or with a selected subset of guide ribonucleic acids (gRNAs) targeting hundreds to thousands of genes. Usually, a project is proposed by a scientific team with extensive knowledge of a specific disease who are involved in the selection of the bespoke gRNA libraries based on the scientific literature, previous experiments, or knowledge graph predictions [15]. The hit genes identified in an arrayed CRISPR screen are then validated in other assays, cell models, and modalities (e.g., short‐interfering ribonucleic acid, inhibitors).

#### 2.1.2 Assay Development

As processes of assay development can vary for different projects in practice, we propose a guideline that has been tested for multiple types of assays. Our recommended steps for assay development start with defining requirements for assay quality through identifying potential CQAs, which can be chemical or biological properties or statistical characteristics. Values of CQAs must be within appropriate specifications, such as within a limit or range, to ensure quality. For in vitro assays, it is necessary to control both signal and noise. Some commonly used CQAs [16] are described below.

For individual signals, precision is often measured by either the sample standard deviation (s) or the *coefficient of variation* (%CV), which is given by $\%\mathrm{CV}=100\%\times \frac{s}{\bar{x}}$, where $\bar{x}$ is the estimated mean. Low s or %CV values are preferable to high values. One might instead consider a *signal‐to‐noise ratio* $\frac{\bar{x}}{\sigma }$, which is essentially an inverse of the %CV, and prefer higher values. At AstraZeneca, depending on the assay, the %CV criterion may be evaluated on controls and/or on the replicates for each evaluated condition.

The dynamic range is also commonly considered a metric for assay quality, as in vitro assays are often optimized within controls that provide biologically relevant high and low signals. For assays that are evaluated in terms of fold change, the dynamic range can be calculated as:

$$\text{Signal}\text{‐}\text{to}\text{‐}\text{background ratio}=\frac{{\bar{x}}_{H}}{{\bar{x}}_{L}},$$

where ${\bar{x}}_{L}$ and ${\bar{x}}_{H}$ denote low and high control means, respectively. For assays that are evaluated by absolute differences, the dynamic range can instead be given as ${\bar{x}}_{H}-{\bar{x}}_{L}$. Large values for the dynamic range are preferable to small values, as this indicates that your assay is more sensitive to changes in the signal of interest.

A popular CQA that incorporates assay variability with dynamic range is given by the Z‐factor (not to be confused with the z‐score or z‐statistic):

$$Z\;\text{factor}=1-\frac{3\left(s_{L}+s_{H}\right)}{\left|\;{\bar{x}}_{H}-{\bar{x}}_{L}\right|},$$

where $s_{L}$ and $s_{H}$ denote low and high control standard deviations.

The *Z* factor value takes one form of the dynamic range as the denominator and multiplies it by a factor that is intended (but fails) to represent the total variability of the dynamic range. The *Z* factor values range from negative infinity to one, where a larger value is better and value of one is best. More recently, authors suggest *strictly standardized mean difference* (SSMD) [17, 18].

$$\text{SSMD}=\frac{\left|{\bar{x}}_{H}-{\bar{x}}_{L}\right|}{\sqrt{s_{L}^{2}+s_{H}^{2}}}$$

While similar in spirit to the *Z* factor, SSMD represents an improvement because its denominator is the true representation of the variability of the dynamic range. SSMD is also a type of signal‐to‐noise ratio for the dynamic range so that large values are better than small values.

If the assay is known to produce an occasional outlier, robust versions of the CQAs may also be considered because it is well known that sample means and standard deviations are easily biased by outliers. For example, the sample mean and standard deviation can be replaced with the median and median absolute deviation (MAD), respectively. In such a case, one might also wish to include the number of outliers detected as a separate CQA.

The acceptance range or limits for CQAs should be determined based on the fitness‐for‐purpose requirements of the assay. At AstraZeneca, we determined CQA limits based on collective experience of different assay modalities, considering false‐positive and false‐negative rates, time, and cost. For example, the criteria for biochemical high throughput screening assays include, but not limited to: SSMD ≥5 and %CV for both low and high controls are lower than 10%. These stringent criteria are necessary because these screens can often been run with no replicates. However, for a more complex cell‐based CRISPR screen with more sources of variability, it is acceptable to have a SSMD ≥4 and %CV between 10% and 20%. A statistician should be consulted for the good practice of assay quality assessment and consider the amount of replication for sufficient power to manage false‐positive and false‐negative rates.

After the CQAs are determined, scientists should explore CPPs, which are variables that are known or suspected to strongly affect CQAs. CPPs are assay variables that should be controlled during hit screening. As shown in the flow chart in Figure 1, understanding the linkage and effect of CPPs on CQAs can be an iterative process requiring more than one round of experimentation. DoE is recommended for testing multiple factors at a time to determine the design space. The advantages of DoE over one‐factor‐at‐a‐time (OFAT) experimentation in assay development have been well established [19, 20]. DoE can be a more efficient use of time and resource and it also enables scientists to use statistical modeling to explore the interaction among factors, nonlinearity, and non‐normal models for better biological insights.

Although experimental execution is sometimes not considered by statisticians, it is important to understand the practical feasibility and limitations to design a suitable experiment for scientists. For instance, biological experiments usually involve handling multiple complex liquids and it is extremely challenging to conduct an experiment on a 96‐, 384‐, or even 1586‐well plate manually with a complete randomized design. The risk of operator error must be balanced with the benefit of randomization. At AstraZeneca, Synthace software (Synthace Ltd., https://www.synthace.com/) is used to link a DoE with the execution of experiments. Scientists can import a DoE file from JMP into a Synthace workflow, program the dilutions of liquids, and generate the necessary files for a range of automated liquid dispensers, including the Dragonfly Discovery (https://www.sptlabtech.com/products/dragonfly/dragonfly‐discovery), Echo Acoustic Liquid Handler (https://www.beckman.com/liquid‐handlers/echo‐525), and CERTUS FLEX (https://www.fgyger.ch/certus‐flex/certus‐flex‐features‐2/?lang=en). This has enabled scientists to use complex but fit‐for‐purpose designs in their experiments.

Statistical models for data analysis should be appropriately selected based on the DoE and CPPs. In brief, one should consider the distribution of the response variable, the scales of continuous variables and whether they require transformation, whether factors are fixed or random, whether effects are linear or nonlinear, and what biological interactions are possible. Data analysis should begin with visualizations of the raw data to observe patterns and identify any errors, outliers, or missing values. After fitting a statistical model, the adequacy of the model should be checked by plotting the residuals and assessing whether the assumptions of the model are valid. The predictions of the model should be plotted to show which regions of the design space meet the desired CQAs, for example using a contour plot, and to show the uncertainty around the predictions (see Ref. [21], for more on interpreting the design space).

In further experiments, the assay can be optimized by exploring the effects of CPPs and their interactions. A response surface design is commonly used at this stage to link the optimal CQA values to a combination of CPPs and derive a design space. The design space is crucial to identifying the range of CPP settings that do not compromise the desired assay quality in future runs.

### 2.2 DoE Software

In the subsequent case studies, we will demonstrate how to design fit‐for‐purpose experiments using JMP [22] software. JMP allows scientists and statisticians alike to design a wide range of experiments in an accessible graphical interface. This includes, but is not limited to, full‐factorial, response surface, space‐filling, mixture, and optimal designs. These designs can then be evaluated in terms of prediction variance across the design space and different levels of CPPs, collinearity in the design matrix, or power to detect significant effects. The chosen design is linked to a statistical model that can be fitted to the results of the experiment via the point‐and‐click graphical user interface. The automated report provides an overview of the model, effects, and the “Prediction Profiler,” which are interactive plots of the predictions. Graphs can be easily generated to check the model adequacy via residuals plots. The JMP “Graph Builder” supports flexible and fast visualizations to explore your data. We will focus on the functionality supported by JMP's graphical interface; however, greater flexibility and functionality is available in the JMP scripting language where R code can also be integrated.

## 3 Case Studies

In this section, we present three examples of QbD in assay development. This includes a description of the assay, an experimental design implemented in JMP, accessible analysis using the graphical interface in JMP, and more advanced analysis in R.

### 3.1 AlphaLISA Assay

AlphaLISA (PerkinElmer Ltd.) is a homogenous bead‐based luminescent amplification assay for quantification of proteins in serum, plasma, supernatants, and cell lysates samples. The AlphaLISA signal, measured in relative light units (RLUs), is based on bringing AlphaLISA donor and acceptor beads in close proximity, enabling a singlet oxygen transfer from the donor bead to the acceptor bead upon illumination at 680 nm. This transfer results in emission of the acceptor bead at 615 nm, which is quantified in an Alpha plate reader. In this sandwich assay, two antibodies raised against the same target protein bind the protein and bring the anti‐species beads (anti‐rabbit acceptor and anti‐mouse donor) together. Multiple antibodies against the target of interest were tested in all possible pairs to determine which antibody pair produced the highest RLU in combination with the antibody and cell lysate concentrations, as given in Table 1.

**TABLE 1:** Factors to optimize in the AlphaLISA assay.

Factor	Levels/range
Antibodies (R = rabbit, M = mouse)	R1–M1, R1–M2, R2–M1, R2–M2, R3–M1, R3–M2
Rabbit antibody concentration (nM)	0.3–3
Mouse antibody concentration (nM)	0.3–3
Cell lysate (×10^6^ cells/mL)	0.25–2

The goal of the AlphaLISA assay development was to determine the conditions that could reliably produce a high AlphaLISA signal while minimizing costs in future assay runs, where costs were driven by higher rabbit and mouse antibody concentrations. The CQA was the AlphaLISA signal and the target was >20,000 RLU, based on the scientists' previous experience with AlphaLISA assays. Note that this study was run without controls (which is not our preference), which is why the chosen CQA only considers signal and not the background or the ratio between the two.

#### 3.1.1 JMP DoE

We created an I‐Optimal design in JMP's “Custom Design” tool. I‐Optimal designs minimize the average prediction variance over the design space, and are well‐suited to the goal of this assay because we want to predict the optimal conditions and have the highest confidence in these predictions. First, we log‐transformed the ranges of the continuous concentration variables (rabbit antibody concentration, mouse antibody concentration, and cell lysate) because they have multiplicative effects. Then, we implemented a 2nd order model to capture main effects, quadratic effects, and interactions. We also included additional quadratic and third‐order interaction effects as a function of Antibodies. The full model is shown below:

$$\begin{array}{c} \text{AlphaLISA}=\beta_{0}+\beta_{1}\cdot \text{Mouse}+\beta_{2}\cdot \text{Rabbit}+\beta_{3}\cdot \text{Cells}\\ \;+\beta_{4}\cdot \left(\text{Mouse}\cdot \text{Rabbit}\right)+\beta_{5}\cdot \left(\text{Mouse}\cdot \text{Cells}\right)\\ \;+\beta_{6}\cdot \left(\text{Rabbit}\cdot \text{Cells}\right)+\beta_{7}\cdot {\text{Mouse}}^{2}\\ \;+\beta_{8}\cdot {\text{Rabbit}}^{2}+\beta_{9}\cdot {\text{Cells}}^{2}\\ \;+\sum_{a=1}^{k-1}{\text{Antibodies}}_{a}\cdot \;\left(\beta_{10a}\right.+\beta_{1a}\cdot \text{Mouse}\\ \;+\beta_{2a}\cdot \text{Rabbit}+\beta_{3a}\cdot \text{Cells}+\beta_{4a}\cdot \left(\text{Mouse}\cdot \text{Rabbit}\right)\\ \;+\beta_{5a}\cdot \left(\text{Mouse}\cdot \text{Cells}\right)+\beta_{6a}\cdot \left(\text{Rabbit}\cdot \text{Cells}\right)\\ \;+\beta_{7a}\cdot {\text{Mouse}}^{2}+\beta_{8a}\cdot {\text{Rabbit}}^{2}+\left.\beta_{9a},\cdot ,{\text{Cells}}^{2},\;\right)+\epsilon \end{array}$$

where *k* is the number of levels in Antibodies, *a* is the *a*th category of Antibodies, which is a dummy variable that indexes the combination of antibodies used, Mouse is log‐transformed mouse antibody concentration, Rabbit is log‐transformed rabbit antibody concentration, and Cells is log‐transformed cell lysate concentration.

**FIGURE 2:**
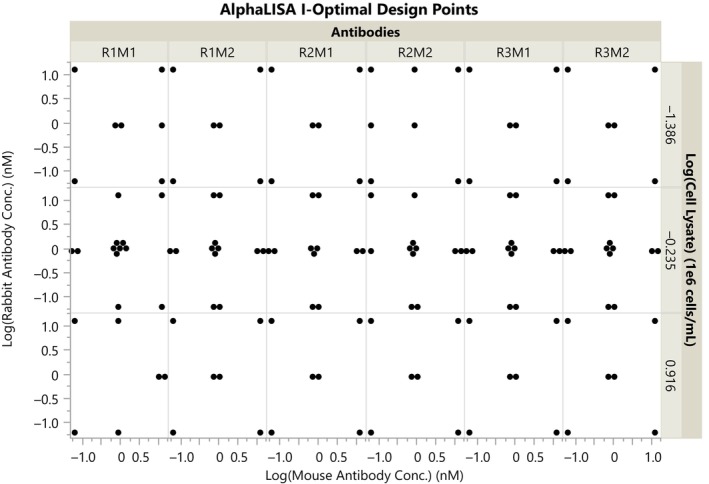
I‐Optimal design points for AlphaLISA assay with 144 runs.

One hundred forty‐four runs were selected as this provided the optimal trade‐off to minimize prediction variance and the reagent costs within the scientists' budget for this experiment. In JMP, a user can readily compare two designs to determine what the marginal decrease in prediction variance would be with additional runs by navigating to “DOE,” “Design Diagnostics,” and then “Compare Designs.” Figure 2 shows how the design points were assigned by the algorithm. Three levels of each continuous variable were included to model quadratic effects, runs were reasonably balanced across factor levels, and additional center points for each antibody condition were included to reduce prediction variance at the center of the design space.

#### 3.1.2 Analysis in JMP

**FIGURE 3:**
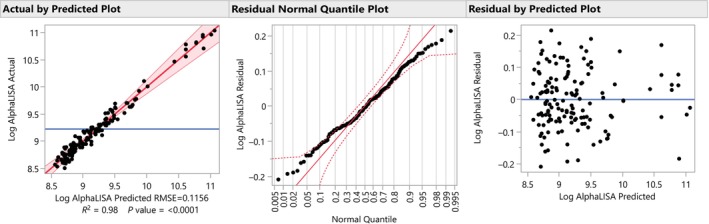
Model adequacy plots.

In this study, the AlphaLISA assay quantified a protein of interest in lysed cells. A log transformation was first applied to the measured AlphaLISA signal to improve linear modeling, as this response typically follows a log‐normal distribution. The statistical model used to generate the design in JMP is retained for the analysis in “Fit Model” and this allows users to run the analysis once the outcome data has been joined to the design. The modeling assumptions of linearity, normality, and equal variance can then be assessed by adding plots in the JMP report. Specifically, the adequacy of the model was checked by plotting the observed versus predicted values, the residuals versus normal quantiles, and the predicted versus residual values (see Figure 3). The JMP *actual by predicted* plot shows that model predictions are very close to the observed log AlphaLISA signal, indicating a good model fit. The *residual normal quantile plot* shows that the residuals are mostly within the boundaries of the diagonal, indicating that they are normally distributed. Finally, the *residual by predicted plot* shows that there is no clear relationship between predicted values and residual variance; this indicates that the residual variance is constant for different predicted values.

**FIGURE 4:**
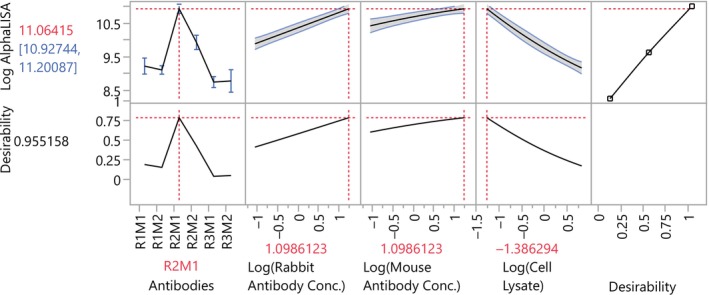
Optimal factor settings for the AlphaLISA assay.

The prediction profiler interactively displays the predicted AlphaLISA (see Figure 4). Users can find the optimal factor settings by enabling the built‐in desirability functions and by maximizing desirability. The desirability function can be set in the DoE interface; here, we chose to maximize the signal and so desirability is simply a linear relationship with log AlphaLISA. However, the desirability function can be modified to meet the goals of the experiment.

To ensure quality in future runs of the assay we generated lower 95% prediction intervals (one‐sided) over a grid of the entire parameter space. This estimates the lower 95% bound of the predicted AlphaLISA signal in future runs for every possible combination of factors in the design. To achieve this in JMP, we first created a new full‐factorial design with five levels of log rabbit antibody concentration from log(0.3) to log (3), 10 levels of log mouse antibody concentration from log(0.3) to log (3), and 10 levels of log lysate concentration from log(0.25) to log (2). This design was concatenated with the data, so that we had a JMP data file with missing responses for the design points originating from the full‐factorial design. We then fitted the model to the data to generate predictions and standard errors of the predictions for the full‐factorial design. The lower 95% prediction interval was calculated as follows:

$$\text{Lower}\;\mathrm{PI}={\hat{y}}_{0}-t_{1-\alpha ,\mathrm{df}}\cdot \mathrm{SE}$$

where ${\hat{y}}_{0}$ is the predicted value, $t_{1-\alpha ,\mathrm{df}}$ is the critical *t* value for *α* (0.05) and degrees of freedom (84), and SE is the standard error of the prediction.

**FIGURE 5:**
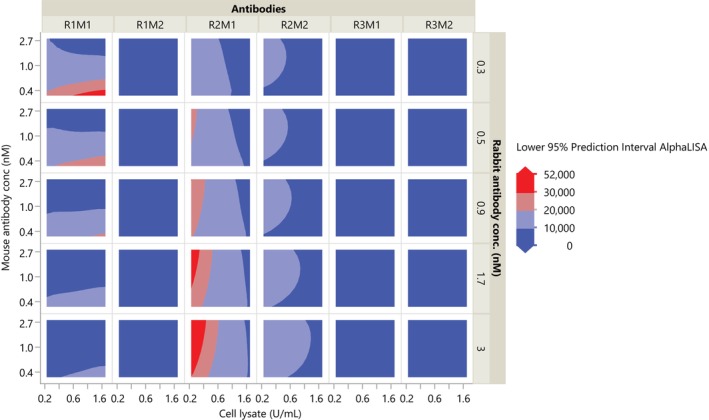
Lower 95% prediction interval for the AlphaLISA assay.

The lower 95% prediction intervals for the parameter grid were visualized as a contour plot using the Graph Builder, as shown in Figure 5. This indicates 95% confidence that the next assay run will produce an AlphaLISA >20,000 if the CPPs fall in the red zones. It is immediately clear that the antibody combinations of R1–M2, R2–M2, R3–M1, and R3–M2 will not produce acceptable quality. For R1–M1, the design space is small, but it uses the smallest amounts of rabbit and mouse antibody concentrations, and therefore cheaper, resulting in the recommendation 0.3 < rabbit and mouse Ab concentration < 0.5 nM and 1 < cell lysate < 2 × 10^6^ cells/mL. For R2–M1, the design space is larger, but uses much larger rabbit and mouse antibody concentrations, resulting in the recommendation 1.7 < rabbit Ab concentration < 3 nM, 0.5 < mouse Ab concentration < 3 nM, and 0.25 < cell lysate <0.4 × 10^6^ cells/mL. Note that the recommendations from this relatively small experiment does not consider the effects of day, experimenter, location, or specific laboratory instruments; an ideal DoE, where resources were not constrained, would include these factors in the design.

#### 3.1.3 Analysis in R

In R, the same log transformations can be made and the same model can be fitted using the code below.


log.AlphaLISA = log(AlphaLISA)


fit = lm( Log.AlphaLISA ~ Ab*(log.rabbit+log.  	mouse+log.lysate)^2 + 


		Ab*I(log.rabbit^2)  	+ Ab*I(log.mouse^2) +


		Ab*I(log.lysate^2) )





**FIGURE 6:**
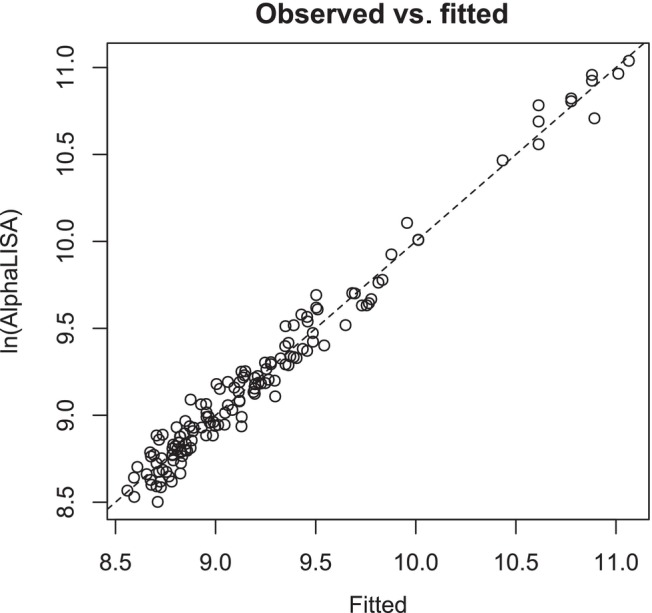
A plot of fitted versus observed values for the quadratic response surface model. The dashed line is the line of unity.

A plot of fitted versus observed values (Figure 6) reveals that the fit is acceptable.

**FIGURE 7:**
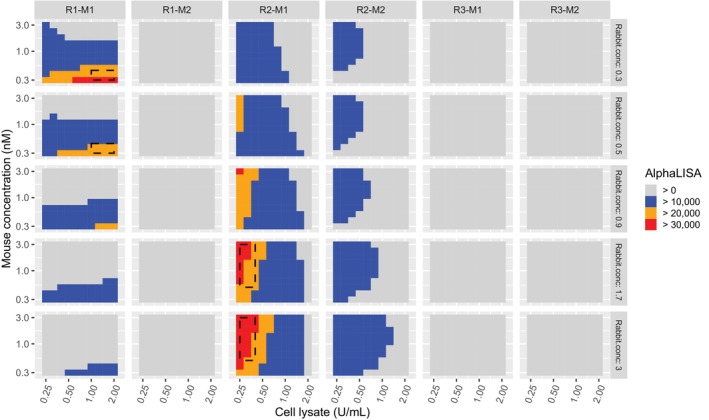
Contour plot of lower 95% prediction interval from I‐Optimal design. A suggested design space is shown by the dashed‐line rectangles.





By constructing lower 95% prediction intervals over a grid of CPP levels, as shown in Figure 7 it may be claimed that we are 95% confident that the next assay run will produce an AlphaLISA >20,000 if the CPPs fall in the orange or red zones. In R, lower 95% prediction intervals for *AlphaLISA* are constructed from the following code.

d.grid = expand.grid(


		Ab=c("R1-M1", "R1-M2",  	"R2-M1", "R2-M2", "R3-M1", "R3-M2"),


		log.rabbit=seq(log(0.3), log(3), length=5),


			log.mouse=seq(log(0.3),  			log(3), length=10),


			log.lysate=seq(log(0.25),  			log(2), length=10)


				)


d.grid = cbind(d.grid,


	predict(fit, newdata=d.grid, interval="prediction", level=0.9))


d.grid[,"lower95p"] = exp(d.grid[,"lwr"])





From Figure 7, we can see the same design space as shown in JMP. Rectangles have been drawn around the recommended design space for future runs. Ultimately, given cost considerations, the investigator chose the R1–M1 design space with lower antibody concentrations.

### 3.2 cAMP Biosensor Assay

Cyclic adenosine monophosphate (cAMP) is a second messenger for numerous G‐protein‐coupled receptors (GPCRs) and changes in cellular cAMP levels thus reflect the biological activity of compounds acting on such GPCRs. The cADDis cAMP biosensor assay (Montanta Molecular) allows for time‐resolved fluorescence analysis of cAMP responses [23]. Cells expressing the GPCR of interest are transduced with BacMAM virus encoding a fluorescent cAMP‐binding protein. When intracellular levels of cAMP change in response to GPCR activation, sensor occupancy follows, and this is reflected in the fluorescence intensity of the cells. To explore assay conditions with sufficient signal to background and the possibility to reduce BacMAM reagent consumption, the CPPs given in Table 2 were tested. The integrated fluorescence signal was measured with the natural GPCR ligand (signal) and DMSO (background) and the CQA is the signal‐to‐background ratio between these two conditions. The goal of the assay was to find CPP settings that achieve a ratio greater than 2.

**TABLE 2:** Factors to optimize in the cAMP biosensor assay.

Factor	Range
Cell number (U/μL)	100–300
BacMam reagent virus (U/μL)	0–4e^7^
Enhancer (mM)	0–5

#### 3.2.1 JMP DoE

**FIGURE 8:**
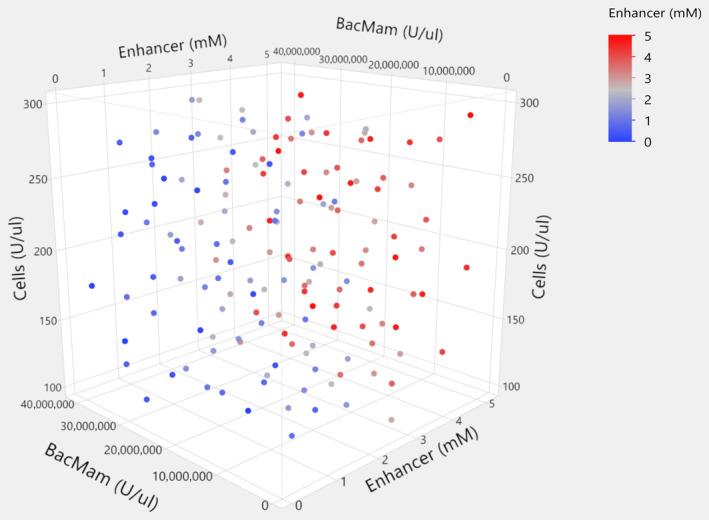
Design points for the 154‐run space‐filling design.

Optimal designs are excellent choices for optimization experiments when trying to maximize the information learnt from minimal runs, as seen in the first case study. However, in this case study the experiment was extremely time‐consuming and additional wells would take little extra time. Therefore, the scientists decided to use all the wells in one 384‐well plate with empty edges to maximize the information learned from one experiment. A 154‐run space‐filling design was constructed to uniformly sample over the entire range of the CPPs and repeated for the signal and background conditions (total runs = 308). The design points were selected using the Latin hypercube method, which evenly distributes the levels of each factor and maximizes the minimum distance between design points (see Figure 8). Such a design allows the calculation of smooth predictions across the design space and facilitates the identification of maxima.

#### 3.2.2 Analysis in JMP

The signal‐to‐background ratio was analyzed using a Gaussian process model in JMP. A Gaussian process is a flexible, nonparametric model that assumes that the data can be represented as a collection of random, Gaussian‐distributed functions [24]. A spatial correlation model was added so that the correlation between the signal‐to‐background ratio for two observations decreases as the distance between the CPPs increases. This approach enables one to identify the optimal CPP settings by fitting smooth predictions across the large number of uniformly spaced levels from our space filling design. As it is a nonlinear and nonparametric method it can be advantageous over linear modeling; however, it is less efficient and requires more data.

**FIGURE 9:**
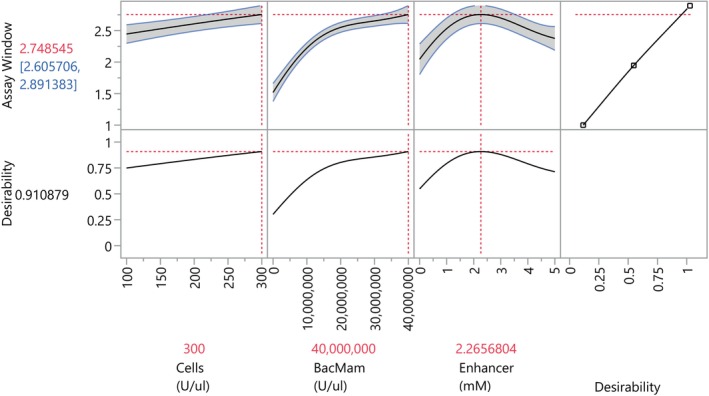
Prediction profiler for the signal‐to‐background ratio.

The highest predicted signal‐to‐background ratio can be found by maximizing the desirability in the prediction profiler (see Figure 9). A signal‐to‐background ratio of 2.75 was achieved for 300 cells (U/μL), 4e7 BacMam concentration (U/μL), and 2.27 enhancer concentration (mM), with 95% confidence that the true mean lies between 2.61 and 2.89.

**FIGURE 10:**
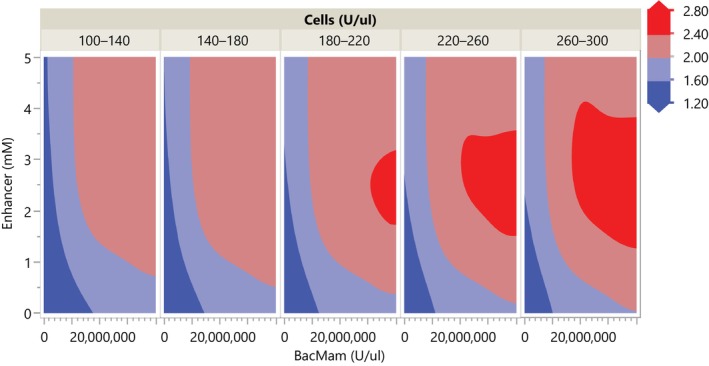
Contour plots of the one‐sided lower 95% confidence interval.

We then calculated lower one‐sided 95% confidence interval of the mean for a grid of the entire parameter space by augmenting the data with a full‐factorial design, as we did in the previous case study. Confidence intervals were calculated as the calculation of prediction intervals for the Gaussian process model was not straightforward in JMP. Contour plots of the lower 95% confidence interval indicate desirable regions of the design space in red where the signal‐to‐background ratio exceeded 2 (see Figure 10). In general, BacMam concentrations greater than 1e7 and enhancer concentrations 2–4 exceeded this threshold, and higher cell concentrations were associated with higher signal‐to‐background ratios. Therefore, using JMP, the design space is 100 ≤ cell number ≤ 300 (U/μL), 1e7 ≤ BacMam ≤ 4e7 U/μL, and 2 ≤ enhancer ≤4 mM; with a target of 300 U/μL cells, 2.5e7 U/μL BacMam, and 2.5 mM enhancer to reduce costs associated with the BacMam reagent.

#### 3.2.3 Analysis in R

Although a Gaussian process model can be fitted in R, to demonstrate a different robust model fit for the space‐filling design data, a thin‐plate‐spline model was implemented, using the gam() function of the mgcv library.


fit = mgcv::gam(log10(Ratio)~s(Cells, BacMam, Enhancer))





In this case, the thin‐plate spline is a penalized smoother for the mean and the errors are assumed to be independent, identically‐distributed normal random variables with mean zero and variance $\sigma^{2}$ [25].

As with the Gaussian process model, this fits smooth predictions across the range of our CPPs. Before fitting, log10 transformations were applied to BacMam, to rescale the BacMam concentration onto a similar scale as the other variables, and to the signal‐to‐background ratio, as the ratio of two log‐normal variables is log‐normal.

**FIGURE 11:**
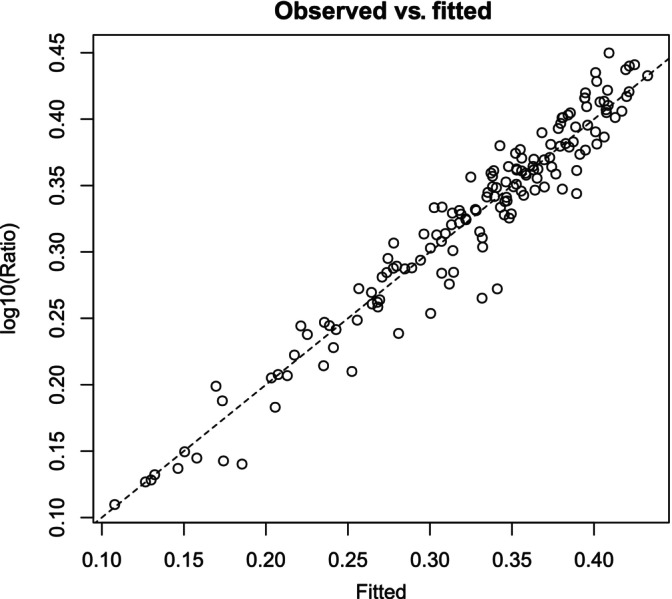
A plot of fitted versus observed values for the gam() model. The dashed line is the line of unity.

A plot of fitted versus observed values (Figure 11) showed that most points were close to the diagonal, indicating a reasonable fit, and that deviations from the diagonal (i.e., residuals) were similarly distributed for different fitted values, indicating equal variance.

**FIGURE 12:**
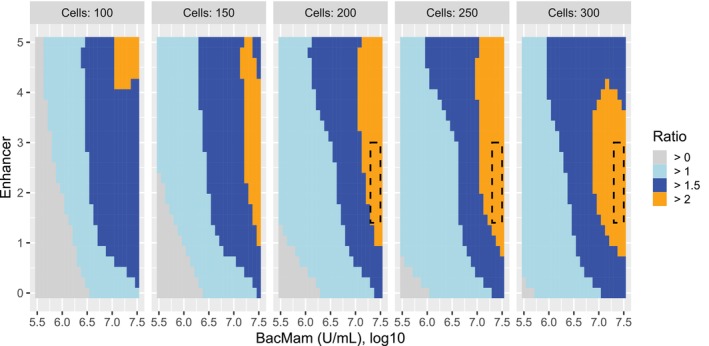
Contour plot of lower 95% prediction interval from gam() model. A suggested design space is shown by the dashed‐line rectangles.





To address whether the next assay run will produce a signal‐to‐background ratio >2, we constructed lower 95% prediction intervals over a grid of CPP levels. In Figure 12, the orange zones indicate at least 95% confidence that the next assay run will produce a signal‐to‐background ratio >2. In R, 95% prediction intervals for the signal‐to‐background ratio are constructed from the following code:

d.grid = expand.grid( Cells=seq(100, 300, length=5),


				BacMam=seq(5.5, 7.5, length=25),


				Enhancer=seq(0, 5, length=25) )


pred = as.data.frame(predict(fit, newdata=d.grid, se.fit=TRUE))


d.grid$lower95p = 10^(pred[,"fit"] - qt(0.95, fit$df.residual)*


					sqrt( fit$sig2 + pred[,"se.fit"]^2 ))





To reduce the use of expensive BacMam reagent, the recommended design space was 200 < cells < 300, 7.3 < log10(BacMam) < 7.5, and 1.4 < enhancer < 3, with a target of cells = 250, BacMam = 7.4, and enhancer = 2. This recommendation could, however, be altered to any hyper‐rectangle that falls in the orange zones in Figure 12 and will likely be a function of assay cost and time. Note, the target CPPs align quite well with the recommendations from the Gaussian process model in JMP, but the design space differs because it depends on the choice of model and on the type of interval. The lower 95% prediction interval is more conservative because these are based on predictions of individual runs, whereas the lower 95% confidence interval is based on the predicted mean.

### 3.3 Arrayed CRISPR Screen

This imaging assay measured a biomarker on the membrane of renal epithelial cells, which classifies them as healthy and functional kidney cells. Images were analyzed with a machine learning algorithm to classify individual cells as either healthy or stressed. The outcome was the proportion of healthy cells in a well and the CPPs were the cells per well (cpw) and concentration of a stressor interleukin‐1β (see Table 3). The goal was to identify a dose–response relationship with the stressor and the CQA was the SSMD of different concentrations relative to no stressor (stressor concentration = 0). The objective was to determine the lowest concentration of the stressor and smallest sample size *N* so that SSMD >5 for a future run with 95% probability. A future run would contain *N* wells for each of two controls of “*no stressor*” and “*stressor*” at the chosen concentration with possible choices *N* = 4, 6, and 8 wells.

**TABLE 3:** Factors to optimize in the CRISPR assay.

Factor	Range
Cell per well	12,500–16,500
Stressor concentration (ng/mL)	0–20

#### 3.3.1 JMP DoE

The number of runs for this experimental design was limited to 64, due to other experimental conditions tested on the plate. The scientists chose two levels for cpw at the upper and lower limits of a range considered to be far enough apart to potentially make a difference, but close enough together to likely result in satisfactory signal. As the dose–response relationship with the stressor was unknown, eight concentrations of the stressor were tested to increase the chances of capturing concentrations near the minimum, maximum, and half‐maximal response. This is a priority in such experiments because missing these key concentrations can lead to poor fit and unreliable predictions. To optimize the coverage of the dose–response relationship, the concentrations selected were approximately evenly spaced on a logarithmic scale. A full‐factorial design (8 concentrations × 2 levels of cpw) was used with four replicates per condition as this would subsequently enable flexibility to model the dose–response relationship collectively or independently with respect to cpw.

#### 3.3.2 Analysis in JMP

**FIGURE 13:**
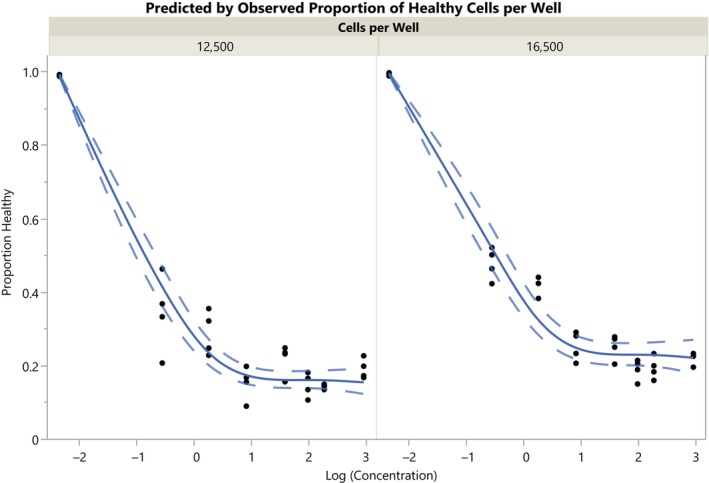
Fitted dose–response curves to proportion of healthy cells per well (solid line) and 95% confidence intervals (dashed lines).

As the response variable (*healthy*) is a proportion bounded between 0 and 1, a logit transformation (logit_healthy = log(healthy/(1—healthy))) was applied to enable linear modeling under the assumption of normally distributed residuals. Log transformations were applied to cpw and stressor concentration (with +0.1 to handle log(0)). The model was fitted with a main effect of cpw and the main, quadratic, and cubic effect of stressor concentration. As before, we can check model fit by plotting the observed by predicted values, the predicted values by the residuals, and the residuals by normal quantiles. We can also examine the fit to the raw observed proportions of healthy cells. To do this, we saved the prediction formula and 95% confidence interval from the analysis report and then performed an inverse logit transform (healthy = 1/(1 + exp(−logit_healthy))) of these columns. We then plotted the observed, predicted and confidence intervals of proportions of healthy cells in Graph Builder (see Figure 13). One can observe that the lowest concentration (corresponding to 0) may be highly influential to the model fit, which could be explored in further assay optimization by testing lower concentrations.

In JMP, we identified a dose–response relationship between the stressor concentration and proportion of healthy cells that showed a good fit to the data. However, the CQA was to determine the SSMD for any given concentration relative to 0 and the power to detect SSMD >5 in future runs with *N* = 4, 6, or 8 wells. Because the statistical distribution of SSMD for a future run is complex, it was not possible to model this using frequentist modeling approaches in JMP that assume a standard distribution.

#### 3.3.3 Analysis in R

To model the distribution of SSMD, a Bayesian approach was taken. In R, the Bayesian computer coding is straight‐forward and fast. The Bayesian version of the linear model may be fitted using the MCMCregress() function of the *MCMCpack* library, given by the following.


fitb = MCMCpack::MCMCregress(logit_healthy~cpw+


		poly(log(conc+0.1), 3, raw=TRUE))





While we could have provided vaguely‐informative prior information for the polynomial coefficients and an informative prior for the variance, we instead used the default prior distributions in MCMCregress(), which are flat priors for the mean‐model parameters and an inverse‐gamma prior with shape = scale = 0.0005 for the variance. To predict future data at a grid of *cpw* and stressor concentrations (*conc*), the following code was implemented.


d.grid = expand.grid( cpw=c("12,500", "16,500"),


	conc=c(0, exp​(seq​(log(0.1), log(20), length=50))),


		N=c(4, 6, 8),


		SSMD=NA, SSMD.prob5=NA )


X.grid = model.matrix(~cpw+poly(log(conc+0.1), 3, raw=TRUE), data=d.grid)





for ( i in 1:nrow(d.grid) ){


	## Posterior distribution of mean at conc = 0 and at conc = d.grid$conc[i]


	mu0 = fitb[,1:5]%*%X.grid[1,]


	mu1 = fitb[,1:5]%*%X.grid[i,]





	## SSMD = (ybar1 - ybar0)/sqrt( s0^2 + s1^2  )


	## Posterior predictive distribution of summary statistics


		## ybar1 - ybar0 ~ N( mu1-mu0,  2*sigma^2/n )


	ybar.diff = rnorm( nrow(fitb), mean=mu1-mu0,


			sd=sqrt(2*fitb[,"sigma2"]/d.grid$N[i]) )





	## (n-1)s0^2/sigma^2 and (n-1)s1^2/sigma^2 ~ chisq(n-1)


	s0.sq = rchisq(nrow(fitb), df=d.grid$N[i]-1)*


			fitb[,"sigma2"]/(d.grid$N[i]-1)


	s1.sq = rchisq(nrow(fitb), df=d.grid$N[i]-1)*


				fitb[,"sigma2"]/(d.grid$N[i]-1)





	ssmd = abs(ybar.diff)/sqrt(s0.sq+s1.sq)





	d.grid$SSMD[i] = median(ssmd) # Median SSMD





	# Probability that future SSMD (sample size N[i]) is > 5.


	d.grid$SSMD.prob5[i] = mean(ssmd >= 5)


}





**FIGURE 14:**
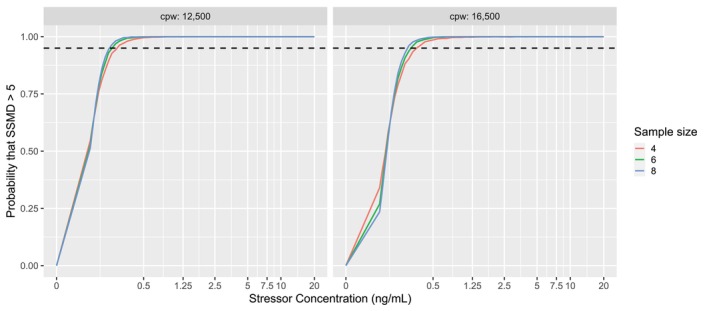
Posterior predictive probability that SSMD >5 for a given sample size (*N*), cells per well (cpw) and stressor concentration. A dashed line was drawn at 0.95 to guide the eye.

A graph of the posterior predictive distribution that SSMD >5 is shown by Figure 14. From the figure, the design space might be constructed from the CPPs for any probability greater than or equal to 0.95. Thus, a potential design space is 12,500 < cpw < 16,500 and 0.5 < stress or concentration < 20 ng/mL with targets of cpw = 14,500 and stressor concentration = 1.0 ng/mL. So long as the assay is operated within the design space, a sample size of *N ≥* 4 should provide a high probability to produce SSMD >5.

## 4 Summary

Assays can be efficiently and robustly developed in preclinical research by implementing QbD principles. In the scoping phase, project aims and prior knowledge are discussed by a multidisciplinary team of scientists, statisticians, and technicians. In assay development, CQAs are defined and CPPs are tested using DoE. Analyses of these experiments identify the design space, which can then be further optimized with subsequent DoEs. The design space is not just critical for optimizing CPPs, it also identifies a region in which the CPPs can be changed intentionally or unintentionally without negatively impacting the desired quality of the assay. In three preclinical case studies, we have shown how DoE and predictive modeling can be practically implemented to rapidly develop high quality and robust assays.

## Conflicts of Interest

J.J., B.Z., X.Z., P.K., A.S., U.K., A.B., and P.H. are employees of AstraZeneca and S.N. was an employee of AstraZeneca at time of study. They may have ownership, options, and/or interests in AstraZeneca stock.

## Data Availability

Data sharing is not applicable to this article as no new datasets were generated or analyzed during the current study.
